# Clinical utility of urinary comprehensive genomic profiling in diagnosing metachronous upper tract urothelial carcinoma: a case report

**DOI:** 10.3389/fruro.2023.1229709

**Published:** 2023-08-09

**Authors:** Paul M. Yonover, Ceressa T. Ward, Brian C. Mazzarella, Kevin G. Phillips, Brad W. Jensen, Vincent T. Bicocca, Kathleen Duffy, Jaden Yonover, Ava Cherry, Trevor G. Levin

**Affiliations:** ^1^ UroPartners/SolarisHealth Partners, Chicago, IL, United States; ^2^ Convergent Genomics, South San Francisco, CA, United States

**Keywords:** metachronous neoplasm, bladder cancer, diagnostic test, DNA mutational analysis, precision medicine

## Abstract

**Introduction and aim of study:**

Metachronous upper tract urothelial carcinoma (UTUC) is a rare yet aggressive malignancy that is often multifocal and invasive at the time of diagnosis. Unfortunately, the rarity of metachronous UTUC results in a paucity of targeted data, as current literature and clinical management of this tumor is largely extrapolated from that of bladder cancer. Urinary comprehensive genomic profiling with the UroAmp assay identifies six general classes of tumor-mutations present in the urine and thus, may aid in detecting UTUC when the limitations of current tools impede definitive diagnosis. We describe the utility of urinary comprehensive genomic profiling in confirming the provider’s suspicion for metachronous UTUC and recommending radical nephroureterectomy.

**Patient case:**

A 68-year-old male with a history of recurrent carcinoma *in situ* (CIS) of the bladder presented to the urology clinic in 2022 for continued surveillance. Abnormal soft tissue thickening surrounding the proximal right ureter, revealed on computerized tomography urography, prompted further evaluation. Selective right upper tract cytology was indeterminate, and urinary comprehensive genomic profiling was ordered to adjudicate. No tumor was visualized on ureteroscopy however the cytologic brush biopsy of the renal pelvis and proximal ureter were positive for urothelial carcinoma (UC) and/or CIS. UroAmp testing identified genomic features associated with high-grade UC, risk of invasion, and a high genomic disease burden.

**Results:**

The patient underwent a right kidney and ureter nephroureterectomy in September 2022. Surgical pathology confirmed non-invasive multifocal urothelial CIS. A postoperative urinary comprehensive genomic profiling in February and May of 2023 detected no evidence of residual disease, consistent with complete resection of the tumor. The provider will continue intensive urinary comprehensive genomic profile monitoring coupled with conventional surveillance.

**Conclusion:**

Urinary measurement of mutated UC genes correlate with disease burden, pathologic grade, and invasion risk and provide clinical utility when reliance on visual confirmation and cytology were not definitive or feasible.

## Introduction

Upper tract urothelial carcinoma (UTUC) accounts for 5-10% of cancers derived from the urothelium (1–3). The incidence of primary UTUC with concomitant bladder cancer is 17%; however, metachronous UTUC following a primary bladder cancer diagnosis occurs in 0.7-5% of patients (2–4). Although rare, UTUC is an aggressive malignancy, which is often multifocal and invasive at time of diagnosis; thus, early, and accurate recognition is critical (1, 5). Current diagnostic tools, such as cytology and cytologic brush biopsy, have documented sensitivity, specificity, and discordance limitations that reduce confidence when recommending guideline indicated interventions, such as radical nephroureterectomy. The Paris System for Reporting Urinary Cytology (6) has created standardized cytologic criteria for diagnosis and improved urologic cytology accuracy, but nephrolithiasis, artifact, and inflammation still make it difficult to obtain a definitive diagnosis, and atypical or suspicious findings are common. The limitations of traditional UTUC evaluation highlight the need for new tools. A noninvasive diagnostic that quantitatively identifies the presence of UTUC could help confidently risk stratify patients, enable guideline adherence, and improve outcomes. Urinary comprehensive genomic profiling uses next-generation sequencing to identify tumor-mutations present in the urine. The UroAmp™ assay (Convergent Genomics, South San Francisco, CA) performs urinary comprehensive genomic profiling to identify six classes of tumor mutations: single-nucleotide variants, gene-level copy-number variants, insertion-deletions, copy-neutral loss of heterozygosity, microsatellite instability, and whole-genome aneuploidy. It was built to identify mutations associated with UC as well as predict molecular grade, disease progression, and recurrence risk (7, 8). Here, we describe the use of urinary comprehensive genomic profiling to confirm metachronous UTUC and reassure the provider’s recommendation for a patient to proceed with a radical nephroureterectomy.

## Case presentation

A 68-year-old non-smoking male with a history of recurrent carcinoma *in situ* (CIS) of the bladder since 2016, hypertension, hyperlipidemia, diabetes mellitus, and coronary artery disease presented to the urology clinic in 2022 for continued surveillance. The patient’s most recent recurrence was 2017, when a surveillance cystoscopy showed a cobblestone appearance of the right trigone and imaging revealed new right hydronephrosis. A transurethral resection and retrograde ureteropyelogram were performed and demonstrated mild hydroureteronephrosis with no filling defect and no specific pathology identified in the upper tract. Pathology from the bladder demonstrated recurrent CIS which was treated with repeat resection followed by induction and a full course of maintenance Bacillus Calmette-Guérin (BCG) therapy. No recurrences had been detected since, and the mild hydronephrosis was stable on imaging.

In April 2022, surveillance computerized tomography (CT) urography showed new abnormal imaging changes in the right ureter suggesting recent passage of kidney stone(s). Repeat CT urography in May demonstrated abnormal soft tissue thickening surrounding the right proximal ureter with intact lumen and drainage. The patient returned to clinic later that month for follow-up cystoscopy, which was negative. Office-based cytology, selective cytology, and retrograde pyelogram were performed, with the pyelogram showing normal upper tract contours and drainage with no filling defect. Cytology of the lower tract was negative for dysplastic cells; however, the right upper tract cytology was “suspicious” for malignancy and dysplastic urothelial cells were suggestive of high-grade UC/CIS. In early June, the physician ordered urinary comprehensive genomic profiling using UroAmp (results described below) to help adjudicate the abnormal cytology. Nine days later, the patient underwent a diagnostic ureteroscopy of the right renal pelvis and proximal ureter with brush biopsies. No visual lesions, either papillary or sessile, were seen in the upper tract; however, selective cytology and cytologic brush biopsy of the renal pelvis and proximal ureter were positive for high-grade UC and/or CIS.

In late June, the patient was seen in clinic, post-operatively, to discuss UTUC diagnosis and recommendations for treatment. Notably, the UroAmp surveillance algorithm reported a high-risk for cancer recurrence and identified genomic features associated with high-grade UC. Urinary comprehensive genomic profiling identified five somatic mutations at variant allele frequencies ranging from 3.2%-10.5%. The mutational profile consisted of single nucleotide variants in *ARID1A* (premature stop codon), *ERBB2* (TCGA hotspot), *ERBB3*, and *TERT* (TCGA hotspot), and a multi-base deletion causing a frameshift in *ZFP36L1*. The following prognostic insight was summarized from available literature: *ARID1A* mutation has been associated with BCG-resistance (9), *ERBB2* and *ERBB3* mutations are more prevalent in non-papillary/CIS tumors (consistent with negative ureteroscopy) (10), and the presence of insertion-deletions are also enriched in high-grade tumors (8). UroAmp further revealed a high genomic disease burden of 70 which alerted the provider that this patient’s mutational intensity is higher than 70% of UC patients previously evaluated.

**Figure 1:**
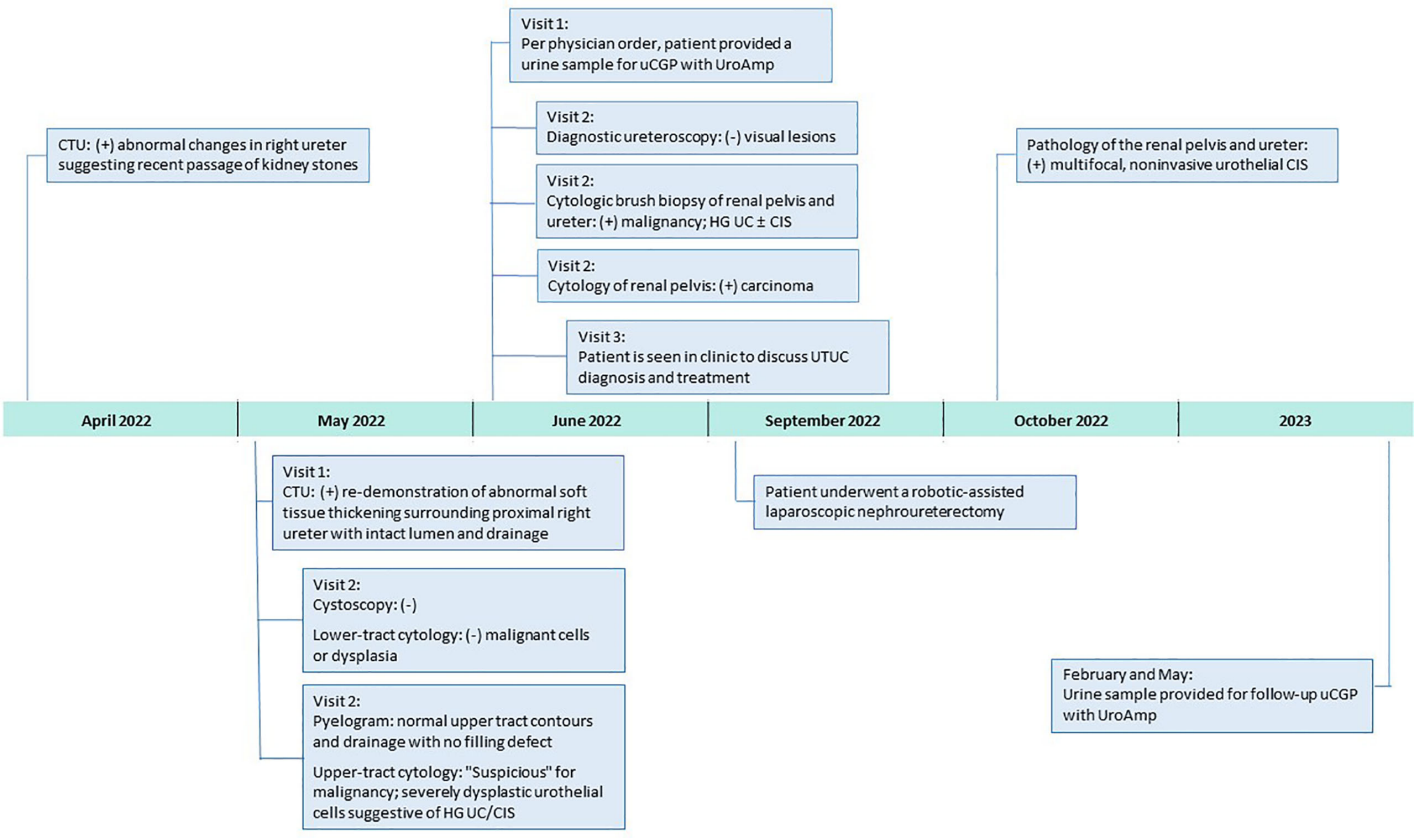
Clinical Course After 2022 Surveillance Visit. CIS, carcinoma in situ; CTU, computerized tomography urography; HG, high-grade; uCGP, urinary comprehensive genomic profiling; UC, urothelial carcinoma.

Based on the combination of cytologic brush biopsy, selective cytology, and urinary comprehensive genomic profiling results, the patient was referred to an academic medical center, and subsequently underwent a right kidney and ureter robotic-assisted laparoscopic nephroureterectomy in September 2022. Surgical pathology confirmed multifocal urothelial high-grade CIS of the right renal pelvis and ureter. No invasive carcinoma was identified, and the renal parenchyma was uninvolved but with mild interstitial inflammation. Surgical margins were free of tumor (**Figure 1**).

**Figure 2:**
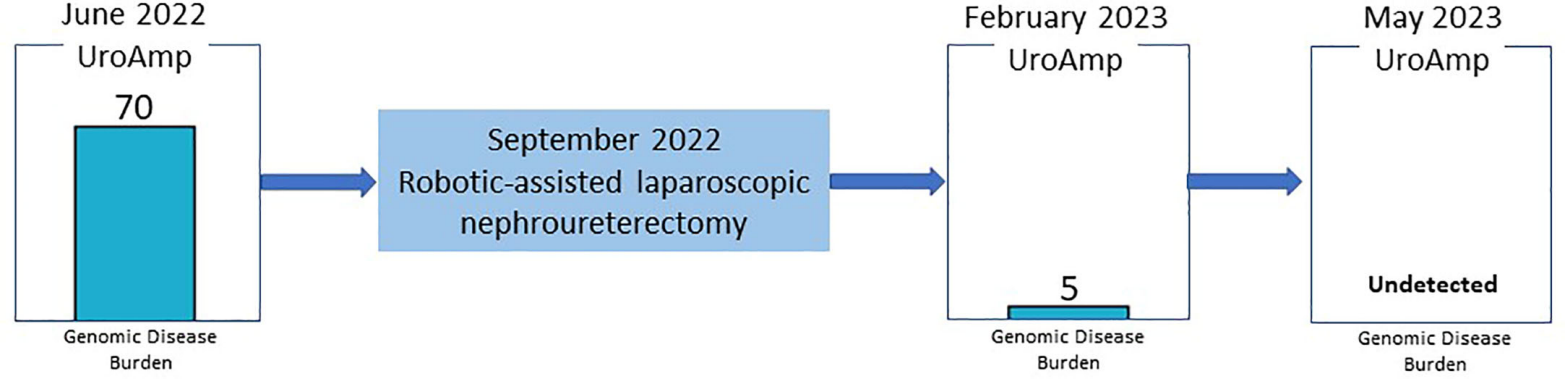
Genomic Disease Burden Before and After Surgical Intervention.

Since the nephroureterectomy, the patient has provided two urine samples for urinary comprehensive genomic profiling during follow-up visits. The first test, in February 2023, reported a genomic disease burden of 5 and found none of the original mutations present. No genomic disease burden was detected in the second test obtained in May 2023 (**Figure 2**). At this time, the provider plans to continue intensive monitoring with urinary comprehensive genomic profile testing coupled with conventional surveillance.

## Discussion

Metachronous UTUC following primary bladder cancer is rare and difficult to confidently identify given quandaries that arise from canonical diagnostic tools. Here, the initial workup showed abnormal soft tissue thickening on CT scan, indeterminate cytology in the presence of negative cystoscopy, negative ureteroscopy, and normal retrograde pyelogram. Follow-up diagnostic ureteroscopy was visually negative and only found positive findings *via* selective cytology and cytologic brush biopsy. These cytologic findings were used to diagnose this patient with UTUC and recommend surgical intervention as per standard of care. There were, however, significant concerns from both the patient and surgeon about choosing radical nephroureterectomy based solely on cytology given its limitations (1, 6, 11, 12). Upper tract urine cytology has grade-dependent specificity for the diagnosis of carcinoma (67%-96%) and poor sensitivity (29%-76%, with most studies around 50%) (1, 6, 7, 13–20). Along with inter-observer variability and a high rate of indeterminate findings, urologists are unable to confidently diagnose UTUC when contemplating radical nephroureterectomy (1, 6, 7, 13–20). Here, the patient’s initial cytology was “suspicious” for malignancy, thus prompting the provider to order urinary comprehensive genomic profiling to adjudicate the indeterminate result and complement any additional findings from the planned ureteroscopy. Notably, urinary comprehensive genomic profiling results encouraged the provider to continue his investigation for potential malignancy.

Given the challenges of staging UTUC, the decision to recommend radical nephroureterectomy is largely based on the diagnosis of high-grade tumor(s) (1). Because histologic evaluation may be impacted by insufficient tissue volume, artifacts, and technique/instrumentation, the use of cytologic brush biopsy is common (1). In a recent study, concordance between brush biopsy and radical nephroureterectomy tissue pathology was 41.1% (grading) and 34.5% (staging), creating insufficient clarity for preoperative planning (21) and highlighting the need for definitive preoperative diagnostic tools to affirm radical nephroureterectomy recommendations.

**Figure 3:**
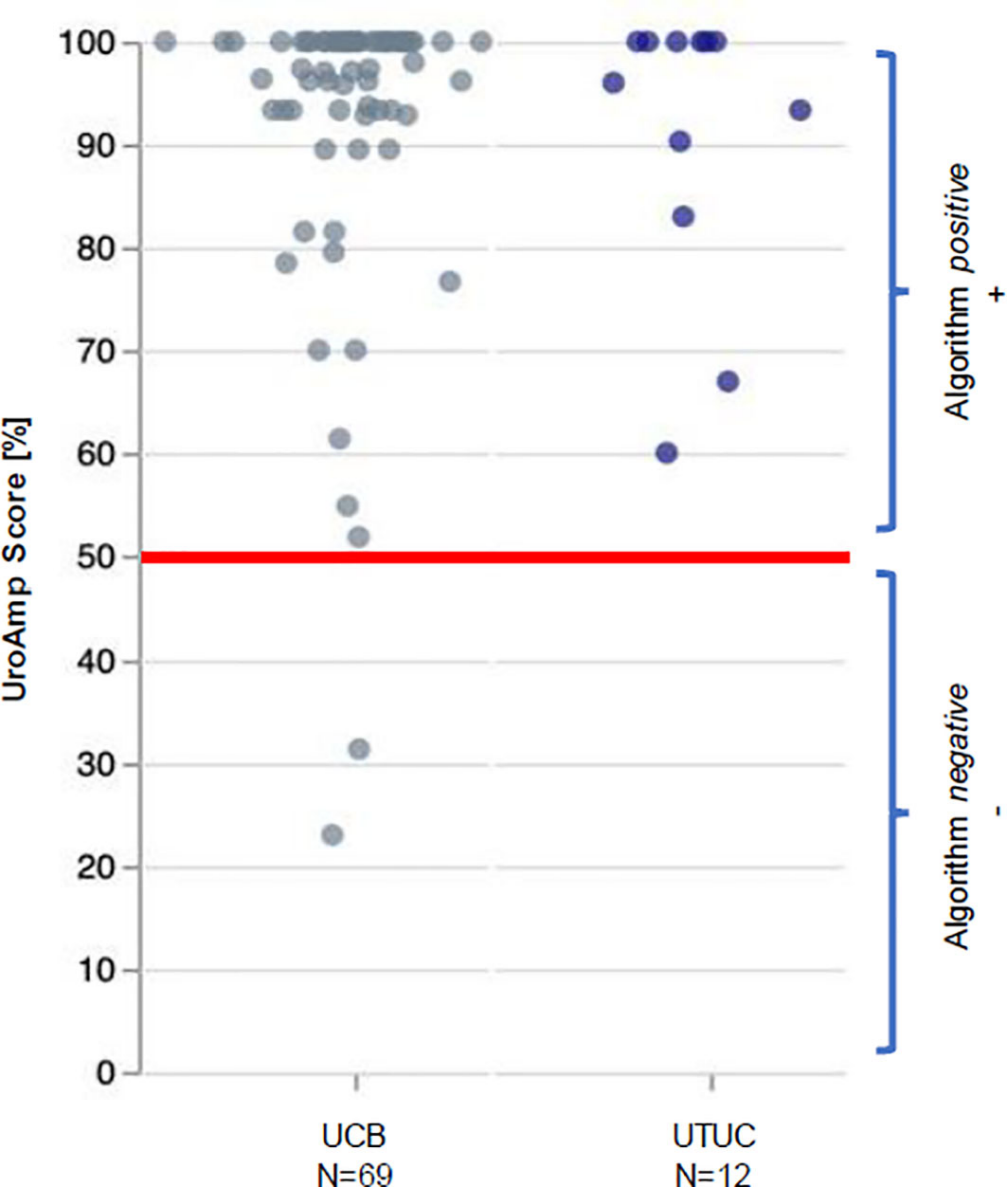
UroAmp Disease Classification of Urothelial Carcinoma. UCB, urothelial carcinoma of the bladder; UTUC, upper tract urothelial carcinoma.

UroAmp’s previously validated diagnostic algorithm has been shown to identify UC with 96% sensitivity and 90% specificity for *de novo* tumors, and a molecular grading algorithm identifies high pathologic grade with 88% positive predictive value and 95% specificity (8). In a cohort of previously analyzed UTUC urine compared to bladder cancer, UroAmp correctly identified 100% of UTUC specimens as disease positive (**Figure 3**; **Supplemental Material**) (8). This patient’s mutation profile included *ERBB2* and *ERBB3* mutations, which promote cell proliferation (22). They were also positive for *ARID1A* mutation, which is associated with high-grade UC and resistance to BCG (9). Found to occur in up to 84% of UC cases, the presence of *TERT* promoter mutations is associated with a higher risk of recurrence (23). *ERBB2/ERBB3* are also associated with non-papillary/CIS tumors and predict responsiveness to ERBB pathway inhibitors (trastuzumab, lapatinib) (9, 22) (**Table 1**). After reviewing the mutations and genomic disease burden provided by UroAmp, the physician was able to confidently recommend radical nephroureterectomy. Notably, when the patient returned to clinic for post-surgical follow-up, UroAmp surveillance testing revealed no evidence of residual disease as none of the tumor’s defining mutations could be detected. Continued surveillance will look for re-emergence of these mutations as evidence of recurrence.

**Table 1:** Major pathways and genomic mutations identified by urinary comprehensive genomic profiling.

Pathway	Gene (mut AA change)	Description	VAF
Proliferation & Immortality	ERBB3 (G582A)	The presence of urinary ERBB3 mutations after surgery is associated with increased risk of recurrence. In muscle invasive disease, ERBB3 mutations are enriched in a luminal expression subtype, which has a favorable overall survival and prognosis compared to basal-subtype tumors.	10.5%
	ERBB2 (S310Y)	ERBB2 mutations are associated with worse prognosis, recurrence, and metastatic potential. ERBB2 may have a higher prevalence in CIS/non-papillary tumors. ERBB2 mutations have FDA-approved drugs available in other tumor types (non-urothelial) and active clinical trials within urothelial carcinoma.	4.4%
	TERT (promoter)	TERT mutations are associated with a 5-fold increased risk of future recurrence in patients with negative cystoscopy.	3.2%
Epigenetic Regulation	ARID1A (Q732X)	ARID1A mutations are associated with worse prognosis, higher grade, and diagnosis at later stage. ARID1A has also been associated with a lack of response to BCG therapy.	8.8%
Translational Regulation	ZFP36L1 (Ser294fs)	Mutations in ZFP36L1 can lead to a loss of function, resulting in diverse overexpression of many proteins through the extended half-life of mRNA molecules. These overexpressions promote uncontrolled cell growth and the development of cancer.	6.6%

BCG, Bacillus Calmette-Guerin; CIS, carcinoma in situ; VAF, variant allele frequency.

In patients with non-muscle invasive UC, the projected 2030 annual costs of $19 billion in the United States will be largely driven by disease recurrence and progression as they necessitate the need for continuous treatment and intensive surveillance (24). Compared to other cancers, management of UC yields the highest economic burden, as rates of recurrence and disease progression remain high (>45%) (25–28). The high cost of care has not translated into improved care, as annual mortality has declined only 2% since 2015 (24). With genomic information, physicians are equipped to make decisions about de-intensifying surveillance, de-escalating therapy in non-responsive patients and/or hastening the time to recommend surgical intervention. These modifications in care have been proven to mitigate high costs (24). Although the financial impact of urinary comprehensive genomic profiling has yet to be determined, access to a genomic profile may allow for cost mitigation strategies as described by Joyce and provide actionable, patient specific data. For our patient, the availability of a urinary comprehensive genomic profile reassured the physician’s decision to proceed with a radical nephroureterectomy which averages $11,793 to $23,235 per patient (29). Alternatively, delaying surgical intervention in a patient with high-grade disease may have led to additional costs related to management of persistent and/or progressive disease.

For patients with non-metastatic high-grade UTUC, radical nephroureterectomy is recommended; however, this procedure has significant perioperative risks, especially in older patients with comorbidities (1, 5, 30). After radical nephroureterectomy, the risk of serious complications is between 11.3-18.2% (5). The rate of perioperative complications secondary to radical nephroureterectomy is limited; however, the most frequently reported include infection (surgical site, sepsis), blood loss requiring transfusion, and renal failure (5, 30, 31). In another alternative clinical scenario, the risk of performing an radical nephroureterectomy where surgical pathology is ultimately negative for malignancy and does not confirm the initial brush biopsy also presents a significant potential healthcare expense, risk for future renal insufficiency to the patient without clinical benefit, and medical liability risk to the treating physicians. Given this patient’s age and comorbidities, an accurate diagnosis of UTUC is prudent when contemplating risks associated with radical nephroureterectomy.

## Conclusion

The diagnosis of metachronous UTUC and recommendation for radical nephroureterectomy were reassured with urinary comprehensive genomic profiling, a new noninvasive diagnostic validated to detect UC with high sensitivity and positive predictive value. Urinary measurement of prognostic genes correlating with high pathologic grade, invasion risk, and genomic disease burden provided clinical utility in this case when reliance on visual confirmation and cytologic brush biopsy were not definitive or feasible. Urinary comprehensive genomic profiling may also prove beneficial in adjudicating indeterminate cytology, detecting UTUC, and providing assessment of grade and invasion risk in scenarios where cytologic brush biopsy is unavailable or insufficient in size for definitive diagnosis, grading, and staging. A prospective study to corroborate findings from this case and the case-controlled cohort is underway.

## Data availability statement

The original contributions presented in the study are included in the article/**Supplementary Material**. Further inquiries can be directed to the corresponding authors.

## Ethics statement

All the procedures involving human subjects described in the study were performed in accordance with the Declaration of Helsinki and were approved by WCG IRB (IRB00000533) under IRB protocol number 120160486. The patients/participants provided their written informed consent to participate in this study. Written and verbal informed consent was obtained from the participant(s)/patient(s) for the publication of any potentially identifiable images or data included in this article.

## Author contributions

PY and CW contributed equally to this work and share first authorship. PY was responsible for conception and design of the study. PY and KP were responsible for the acquisition of data. CW and BM were responsible for drafting the manuscript. All authors contributed to the article and approved the submitted version.
