# Supplementary material for: Clinical utility of urinary comprehensive genomic profiling in diagnosing metachronous upper tract urothelial carcinoma: a case report
Source: Front Urol. 2023 Aug 9;3:1229709. doi: 10.3389/fruro.2023.1229709 (PMC12327264; doi:10.3389/fruro.2023.1229709)
Supplement: Supplementary file 1 [file DataSheet_1.pdf]

Supplemental Material

Objective:
To evaluate the performance of the CLIA-validated UroAmp assay (Convergent Genomics) in identifying upper tract urothelial carcinoma (UTUC).

Methods:
Urinary comprehensive genomic profiling was performed on 69 specimens from individuals with *de novo* pathology-confirmed urothelial carcinoma of the bladder (UCB) and 12 urine specimens from individuals with *de novo* pathology confirmed UTUC. Urine DNA was sequenced, comprehensively profiled across 60 actionable genes to detect six general classes of mutations and ascribed to a disease classification as predicted by a machine learned algorithm.

All the procedures involving human subjects described in the study were performed in accordance with the Declaration of Helsinki and were approved by WCG IRB (IRB00000533) under IRB protocol number 120160486. Subject informed consent was obtained verbally, prior to participation, in accordance with the minimal risk designation of this study.

Results:
UroAmp correctly identified 97% (67/69) of UCB and 100% (12/12) of UTUC specimens as disease positive.
